# Public Sentiment and Discourse on Domestic Violence During the COVID-19 Pandemic in Australia: Analysis of Social Media Posts

**DOI:** 10.2196/29025

**Published:** 2021-10-01

**Authors:** Kim Usher, Joanne Durkin, Sam Martin, Samantha Vanderslott, Cecilia Vindrola-Padros, Luke Usher, Debra Jackson

**Affiliations:** 1 University of New England Armidale Australia; 2 Oxford Vaccine Group University of Oxford Oxford United Kingdom; 3 University College London London United Kingdom; 4 Griffith University Goldcoast Australia; 5 University of Sydney Sydney Australia

**Keywords:** COVID-19, domestic violence, social media, Twitter, sentiment analysis, discourse analysis, keyword analysis, pandemic, sentiment, public health, public expression

## Abstract

**Background:**

Measuring public response during COVID-19 is an important way of ensuring the suitability and effectiveness of epidemic response efforts. An analysis of social media provides an approximation of public sentiment during an emergency like the current pandemic. The measures introduced across the globe to help curtail the spread of the coronavirus have led to the development of a situation labeled as a “perfect storm,” triggering a wave of domestic violence. As people use social media to communicate their experiences, analyzing public discourse and sentiment on social platforms offers a way to understand concerns and issues related to domestic violence during the COVID-19 pandemic.

**Objective:**

This study was based on an analysis of public discourse and sentiment related to domestic violence during the stay-at-home periods of the COVID-19 pandemic in Australia in 2020. It aimed to understand the more personal self-reported experiences, emotions, and reactions toward domestic violence that were not always classified or collected by official public bodies during the pandemic.

**Methods:**

We searched social media and news posts in Australia using key terms related to domestic violence and COVID-19 during 2020 via digital analytics tools to determine sentiments related to domestic violence during this period.

**Results:**

The study showed that the use of sentiment and discourse analysis to assess social media data is useful in measuring the public expression of feelings and sharing of resources in relation to the otherwise personal experience of domestic violence. There were a total of 63,800 posts across social media and news media. Within these posts, our analysis found that domestic violence was mentioned an average of 179 times a day. There were 30,100 tweets, 31,700 news reports, 1500 blog posts, 548 forum posts, and 7 comments (posted on news and blog websites). Negative or neutral sentiment centered on the sharp rise in domestic violence during different lockdown periods of the 2020 pandemic, and neutral and positive sentiments centered on praise for efforts that raised awareness of domestic violence as well as the positive actions of domestic violence charities and support groups in their campaigns. There were calls for a positive and proactive handling (rather than a mishandling) of the pandemic, and results indicated a high level of public discontent related to the rising rates of domestic violence and the lack of services during the pandemic.

**Conclusions:**

This study provided a timely understanding of public sentiment related to domestic violence during the COVID-19 lockdown periods in Australia using social media analysis. Social media represents an important avenue for the dissemination of information; posts can be widely dispersed and easily accessed by a range of different communities who are often difficult to reach. An improved understanding of these issues is important for future policy direction. Heightened awareness of this could help agencies tailor and target messaging to maximize impact.

## Introduction

COVID-19 has affected millions of people across the globe [1]. While the rates of COVID-19 in Australia have been low compared to the rest of the world, Australian people have experienced distress due to the nature of the infection and transmission routes, which have led to many societal and lifestyle changes associated with government attempts to contain the infection [2]. Social distancing regulations imposed to contain the spread of the infection have affected the social, psychological, and economic well-being of many Australians [3-5].

Studies have found that women experienced higher levels of psychological distress during the pandemic when compared to men [6-9]. Younger women in Australia reported higher levels of stress during 2020 compared to older women, with 1 in 4 women aged 25 to 31 years reporting being very or extremely stressed [10]. Reasons for these higher levels have been associated with caring roles and responsibilities [7], linked to homeschooling efforts, job insecurity and financial burden [10], and domestic violence [11,12].

Social media plays a significant role in the dissemination of health information [13], particularly during times of public health crisis [14], and can significantly bolster disaster management communication [15]. Communication during the COVID-19 pandemic has generated more reliance on online platforms such as Twitter, Facebook, and Instagram [16]. Social media can also accelerate the expression of feelings about public events [13,17-19] and social media platforms such as Twitter, blogs, and other platforms are ideal places to quickly receive news and express opinions in times of crisis such as the current pandemic [18,20-22]. As a real-time network, social media offers users the ability to communicate using both public and private messages. Since the beginning of the pandemic, people have used social media sites to express their opinions and share information about the COVID-19 pandemic and related issues. It has been suggested that reliance on social media platforms such as Twitter will continue to grow as long as social distancing measures are used by governments to contain the spread of the virus [23]. Numerous studies have already been conducted to understand the public response to issues related to COVID-19 [18,19,24-26], and social media provides researchers with an opportunity to study the role it plays in the current global health crisis.

The measures introduced across the globe to help curtail the spread of the coronavirus have led to the development of a situation labelled as a “perfect storm” to trigger a wave of domestic violence related to psychological and economic pressures, as well as negative coping mechanisms such as alcohol and other drug use [27,28]. In Australia, alcohol sales rose more than 36% as social distancing measures were implemented [29]. During this period of social isolation, reports of an increase in domestic or intimate partner violence have been heard internationally [27,30-33], reflected in a similar increase in calls to domestic violence hotlines [34]. Similar trends have been reported in previous pandemics [35,36]. In addition, there have also been reports of homicides associated with domestic violence since stay-at-home measures were introduced [37,38]. The pandemic also meant there were fewer opportunities for people experiencing domestic violence to seek help [5,39]. Previously, Twitter hashtags have been examined to determine the nature of domestic violence [18,40,41]. In this paper, we present the findings of a sample of geotagged Australian posts from across different social media platforms, as well as an analysis of users’ public emotional responses to domestic violence during the COVID-19 pandemic. The aim of this study was to understand more personal, self-reported experiences, emotions, and reactions toward domestic violence in Australia that were not always classified or collected by official public bodies during the pandemic.

## Methods

### Data Collection

An initial general Boolean search (Multimedia Appendix 1) was conducted to obtain a broad overview of the main domestic violence topics related to COVID-19. The research included an analysis of social media and news posts via the media monitoring software Meltwater [42] by using advanced Boolean search terms, agreed upon by 5 researchers (KU, JD, SM, SV, DJ), to collect an initial sample of posts related to domestic violence from the Australian region and during the first 6 months of the COVID-19 pandemic (January 1 to June 31, 2020). Posts were collected from January 1 to gather all mentions of the pandemic from the early stages of reports of COVID-19 (including the first reported case in Australia in late January 2020), and any pre-emptive discussion with regards to the effect of localized lockdowns on domestic home situations in Australia.

Overall, the initial search found a total of 137,300 posts that mentioned domestic violence in general across social media and news media. Within these posts, domestic violence was mentioned an average of 300 times a day. There were 90,200 tweets, with 40,300 news posts, 7000 blog posts, 250 forum posts, and 50 comments. Within this exploratory search, we found that with our simple Boolean, despite it being within the range of the start of the COVID-19 pandemic, 35% of all posts did not refer to the context of the pandemic. While this meant there was a need to refine the Boolean with more COVID-19–specific terms and hashtags, we managed to collect specific colloquialisms, slang, and Australian-specific terms linked with domestic violence as experienced in the region, which we then added to a more specific Boolean search. These included terms such as “DFV” (domestic family violence), “DV” (domestic violence), and “VAW” (violence against women). Other terms specific to the Australian region included “domestic abuse,” “family violence,” “intimate partner violence,” “gender violence,” “spousal abuse,” or “spousal violence”—which were found to be used online more often within the Australian region, than, for example, in the United Kingdom or the European Union.

Once an overview of the data was established, we conducted a second, more focused Boolean search (Multimedia Appendix 1), which included keywords and hashtags found in the initial data sample. Usage referred to English-language posts relating to domestic violence from the Australian region and included specific terms related to the first year of the COVID-19 pandemic (January 1 to December 31, 2020).

### Data Analysis

While Meltwater, a standard media monitoring tool, was used to collect social media and news posts, we were aware of the difficulties of accurately assessing and classifying patterns of discourse, sentiment, and emoji-related analysis within the correct context or perspective of the topic under investigation [43]. We then conducted additional discourse and emoji analysis using InfraNodus, a text network analysis software [44,45], to measure themes and patterns occurring in discussions around domestic violence, and to analyze connections between subtopics that linked different types of conversations and word clusters together in order to identify similarities in behavior. Analysis of the sentiment of text and emojis shared within posts was based on the internal InfraNodus emoji sentiment lexicon [46] and sentiment analysis lexicon [47].

While these embedded tools were useful for a broad overview of sentiment, to obtain a better understanding of sentiment from the specific context of domestic violence, we drew on previous research [48] by creating a “manual sentiment framework” (Multimedia Appendix 2). We set up our own framework with definitions based on our analysis to “reannotate” a subsample of posts using this more specific, carefully constructed, qualitative framework. The sentiment of posts shared was measured in terms of differences in attitudes toward dealing with domestic violence within the context of the pandemic (Multimedia Appendix 2). Posts were classified as positive toward the handling of domestic violence issues if, for example, they agreed with official or government policies and actions around this topic. Posts were marked as negative if they contained negative attitudes or arguments against official or government policies and actions on domestic violence during lockdowns or during the pandemic in general, or shared bad experiences, or, for example, discouraged the following of official guidelines and actions due to concerns over the effectiveness of these measures. Posts were marked as neutral if they contained only a general statement, with no expression of sentiment or opinion.

Posts were then coded into predefined categories to create the final data set. Intercoder reliability comparison queries were assessed to ensure accuracy using the processes and scripts demonstrated by Kummervold et al [49], with an accuracy F-score of 0.76. Once we identified the key themes, we selected individual posts for textual analysis, drawing out specific issues for a qualitative interrogation of topics of concern. We draw on Lewis et al [50] for our approach to analysis, treating empirical materials not only as data that are freestanding but as an “empirical trigger” to guide us in describing and analyzing research topics. In addition, we employed constructivist grounded theory [51] in the interpretation of empirical material, following an iterative and reflexive process. Semantic discourse and topic analysis were used to understand frequently used keywords and topics of concern [52].

### Ethics

Ethical issues related to internet research necessitate the need for researchers to carefully consider relevant guidelines to determine whether ethical approval and informed consent are required [53]. The research team discussed the proposed study with the Chair of Human Research Ethics Committee at the University of New England, Australia, who assessed the request and determined no formal ethics approval was required. While this study was beyond the scope of the human ethics committee, we adhered to the principles of ethics: beneficence, nonmaleficence, autonomy, and justice [54]. The appropriateness and benefit of conducting research during the pandemic was considered against the prospect of the results of this research benefiting a wider community [55]. We further considered the mitigation of the risk of harm to participants. In the case of this research, only publicly available tweets were included in the data set. Social media usernames were removed from the data samples, while within the manuscript, only summaries of tweets were used. No direct or easily traceable quotes have been included. All of these measures are in line with best practices [48,49,56,57]. Data were collected from and analyzed via secure encrypted servers via the Meltwater and InfraNodus platforms. Data were subsequently managed on a secure, password-protected drive only accessible by the research team.

## Results

**Figure 1:**
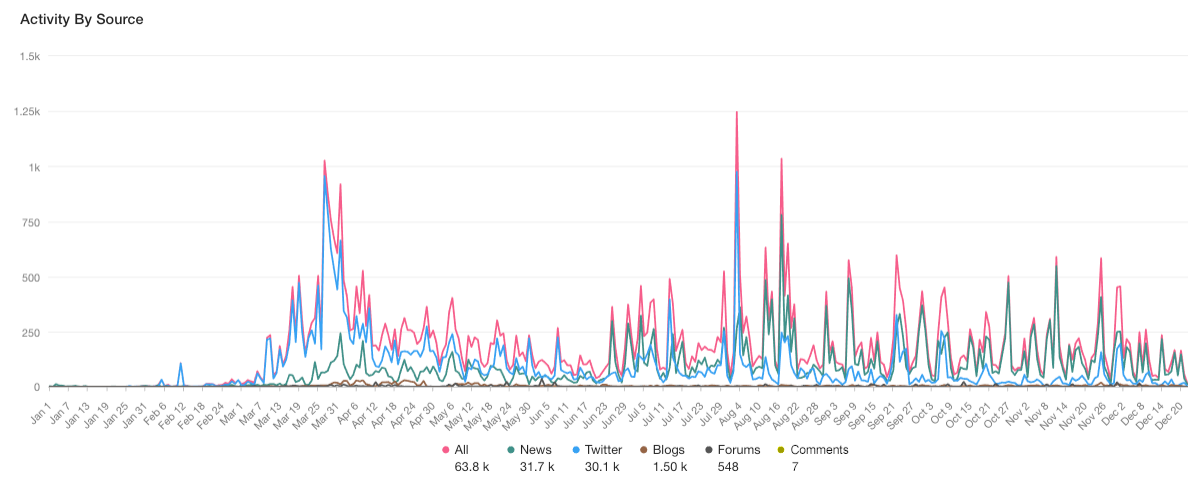
Social and mass media mentions of domestic violence in Australia (January 1, 2020, to December 22, 2020).

In the final data sample, overall, there were a total of 63,800 posts across social media and news media (Figure 1). Within these posts, our analysis found that domestic violence was mentioned an average of 179 times a day. There were 30,100 tweets, 31,700 news reports, 1500 blog posts, 548 forum posts, and 7 comments (posted on news and blog websites).

### Demographics

Within the final data sample, it was found that only a smaller sample of 10,200 (out of 30,100) tweets had demographics data. This is indicative of the complexity of demographically and geographically tagged data [58], which we discuss further in the Limitations section. However, analysis of this smaller sample found that those who identified as female comprised 57.2% (n=5834) of the people posting about domestic violence in Australia during the 2020 pandemic, while those who identified as male accounted for 42.8% (n=4365). The age range of posters was as follows: 18-24 years (n=2029, 19.9%), 25-34 years (n=4386, 43%), 35-44 years (n=2519, 24.7%), and 45-54 years (n=948, 9.3%). While the main family status of posters was listed as parents (n=948, 91.1%), 6.3% (n=642) identified as married, with the rest identifying as senior or single.

On Twitter, the majority of Twitter mentions of domestic violence were retweets (n=17,600), followed by quoted (commented on) tweets (n=7130) and replies (n=1180). This conversation related to engagement among 11,800 Twitter users, centering around 4250 original tweets during this period (January 1 to December 22, 2020). Overall, based on the follower count of each of the users engaging with tweets, the potential reach and impressions of these conversations (possible number of people who may have read these messages) was 177 million.

### Sentiment by Source

While retweeted and reposted news stories (without any additional commentary or human sentiment added) were classified as neutral across networks, the overall sentiment toward the topic of rates of domestic violence was negative at 38% (n=24,264), with just 7% (n=4469) of all posts across social media holding positive keywords. Forum discussions held the most negative sentiment at 50% (n=274), closely followed by reported news items with additional commentary (n=14,265, 45%), tweets (n=12,040, 40%), and blog posts (n=405, 27%).

### Key Drivers of Sentiment

We found that a mixed range of sentiment was used in how domestic violence was discussed on social media and in news articles, as well as expressed in peoples’ reactions to events as they retweeted/reposted or quoted original posts and articles discussing or describing worry or reactions to how domestic violence was handled in Australia during the pandemic and the various lockdowns.

The majority of negative-to-neutral sentiment (Multimedia Appendix 2) centered on the sharp rise in domestic violence during different lockdown periods of the 2020 pandemic, with a focus on gender-based violence, sexism, and worries that a financial crisis and stress on overall services was driving up inequalities and creating a crisis of job insecurity and social pressures that were in turn fueling domestic violence. Neutral-to-positive sentiment centered on the praise of efforts raising awareness of domestic violence, a focus on both violence in immigrant families and stress on nonvisa versus visa holders, help with family-based violence, as well as the positive actions of domestic violence charities and support groups in their campaigns to help isolated victims in both mainstream and Aboriginal communities.

Further analysis of trending negative and ambiguous keywords (posts or articles with a mix of negative and positive terms) showed mixed sentiment, with calls for a positive and proactive handling (rather than a mishandling) of the pandemic. There were calls for a proactive and overdue review of family law, the use of family courts, an easing of the pressure of bankruptcies, and much better access to general practitioner surgeries and help via hospitals to make sure that the knock-on effects of issues that might lead to domestic and family violence could be better handled. More negative sentiment used the term “domestic violence” instead of family violence, with references to an ongoing crisis of domestic violence during various localized lockdowns around Australia, restrictions of access to needed help from the police and emergency services who were diverted elsewhere during the initial lockdowns, and an increased amount of pharmacies and frontline health care workers noticing more women with suspected domestic abuse injuries, as well as worry about pressures on mental health.

### Emoji Analysis

**Figure 2:**
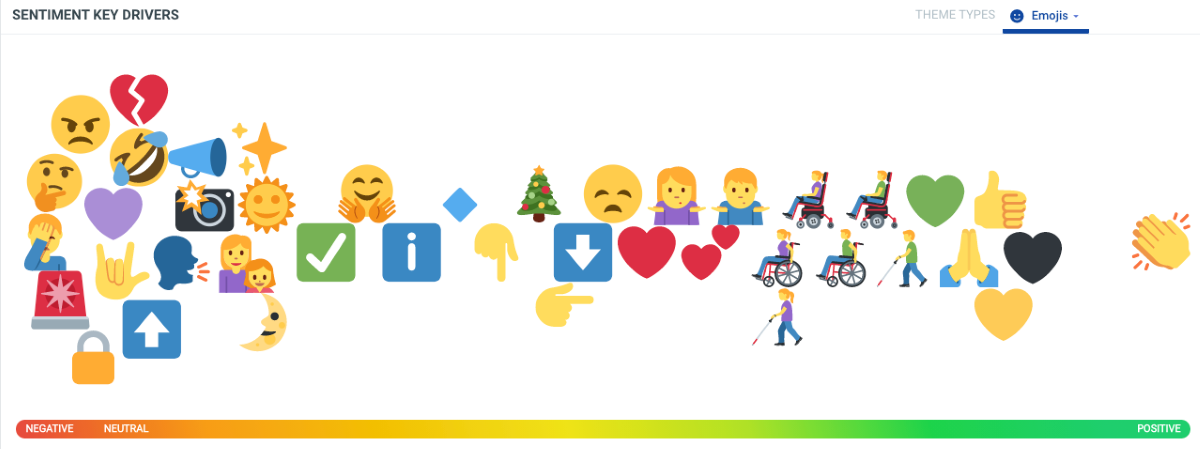
Collection of top emojis used in relation to tweets mentioning domestic violence and the discourse behind them.

The majority of negative emojis used on social media within discussions regarding domestic violence (Figure 2) involved symbols of anger, sadness, annoyance, and disbelief; reports of increased callouts to emergency services (siren emojis); a call for peace; and heartbreak at the rise in instances in family and gender-based violence (older and younger female emojis). The thinking and hand taking notes emojis were used to denote the need to think seriously about taking measures to protect the emotional and mental health well-being of those experiencing domestic and family violence. The sun, stars, and moon emojis conveyed the need to make services accessible to victims both during the day and night. Negative-to-neutral emojis were connected to the arrow and pointing finger emojis that were related to pointing toward links to articles, images, or other resources shared. The open-handed hug emoji was used to denote a show of support to those experiencing domestic violence. The sad or anxiety-face emoji was linked to worry about a surge in domestic violence around Christmas time (Christmas tree emoji), with offers of help, love, and support shared via links and messages (heart emojis). As part of the International Day of People with Disability, there was also a request to raise awareness around the plight of people with physical and visual disabilities (wheelchair and visibility cane emojis). Positive emotion was also represented by variations of different-colored heart emojis, as well as the clapping hands emoji, which symbolized both praise of a new $25 million United Nations emergency fund to tackle violence against women during the pandemic, as well as a celebration of the publication of a research paper that investigated risk and protective factors for family violence both during and after the COVID-19 lockdowns) [59]. While emojis were used across social media platforms (eg, tweets, blogs, forums, and other posts), which is to be expected, no emojis were found to be used within original news media articles.

### Hashtag Analysis

On social media and in news articles, outside of the main COVID-19 hashtags (#covid19 and #coronavirus or #lockdown), among the top 20 corelated hashtags regarding the topic of domestic violence in Australia were ones that focused specifically on the Australian experience (#covid19au or #covid19aus to represent COVID-19 Australia; #covid19vic or #covidvic to represent the experience of COVID-19 in the state of Victoria; #auspol to represent Australian politics; and #springst to represent news and discussion of politics in the Australian state of Victoria [the Victorian Parliament is located on Spring Street]). There were also hashtags linked to colloquialisms for domestic violence. These included #DV as a shorthand for “domestic violence,” #DFV for “domestic family violence,” as well as #VAW for “violence against women.” The hashtag #thedrum was also used in relation to the Australian topical media/news company “ABC The Drum,” which covered a host of topical pieces discussing the rise in domestic violence in Australia during the pandemic. More direct terms like #DomesticViolence and #DomesticAbuse were also popular, with #DomesticViolence being more prevalent (3700 mentions) than #DomesticAbuse (610 mentions).

Negative-to-ambiguous hashtags centered around posts discussing increased rates of stress, mental health, and femicides or deaths linked to substantial increases in domestic violence during lockdown, with specific mentions of New South Wales (#NSW, #nswbudget), as well as the feminist hashtag #MeToo, references to ongoing conversations in Australian politics discussing a need for changes in family law to better protect families experiencing trauma related to domestic family violence. Various corelated hashtags were also linked to wider campaigns focused on supporting victims, such as #ifyoucouldseewhatisee, #SexNotGender, #StaySafeStayOpen, #ChildPoverty, #GenderBasedViolence, #noexcuseforabuse, #ListentoVoices, and #Indigenous. More positive and ambiguous-to-positive hashtags used in campaigns included #LGBTIQ, #trans, #StopDV20, #WAFV2020, #16DaysOfActivism, and #justiceforall.

## Discussion

### Principal Findings

Government-regulated COVID-19 lockdowns occurred throughout 2020 and 2021 in Australia and internationally. Through the use of sentiment and discourse analysis, we identified negative or neutral sentiment centered on the sharp rise in domestic violence during different lockdown periods in Australia. Neutral-to-positive sentiment centered around praising efforts to raise awareness of domestic violence, as well as the positive actions of domestic violence charities and support groups in their campaigns. We identified a high level of public discontent related to the rising rates of domestic violence and the lack of services during the pandemic.

Social media and news media are an important mechanism for discussing and forwarding information about domestic violence and available services [60]. They represent an important avenue for dissemination of information that can be widely dispersed and easily accessed by a range of different communities who are often difficult to reach. Increasingly, Twitter has been utilized to gain insights into public health outcomes, perspectives, and behaviors during the COVID-19 pandemic [61-64].

Our study examined public discussions and shared articles about domestic violence during the initial period of COVID-19 in Australia during 2020. The study contributed to our understanding of public sentiment about domestic violence. It indicated a high level of public discontent related to the rising rates of the violence and the lack of services during the pandemic. Previous studies are consistent with these findings [19,60]. The high rate of negative posts and articles related to domestic violence and COVID-19 supports recent reports and publications that have identified the increasing problems related to domestic violence during the COVID-19 pandemic stay-at-home orders [5,32,39,65-68]. The high rate of negative sentiments about domestic violence could have a negative impact on women experiencing domestic violence as women often feel blamed or blame themselves for the violence [69,70]. In this study, more negative sentiments were related to domestic violence than family violence. This may be related to an understanding of the term and indicates that family violence may be understood as inclusive of children as well as women. The language chosen for social media and news posts can offer insight into issues of what type of violence has been most prevalent and the emotional experiences and reactions to violence.

Emojis are useful for expressing ideas and sentiments on social media, and their use to communicate issues related to public health are not new [16]. In this study, they represented and symbolized the deeply emotional aspects of domestic violence and the vulnerability experienced by women. In a study by Al-Rawi et al [16], more negative emojis were used for women’s concerns than those of men. Negative emojis in this study were used to express anger, sadness, annoyance, and disbelief, while open-handed hug emojis were used to offer support to people who discussed their personal experiences of domestic violence on social media. The sad or anxiety-face emoji was linked to worry about a surge in domestic violence around Christmas time (Christmas tree emoji), with offers of help, love, and support shared via links on social media (heart emojis). As part of the International Day of People with Disability, there was also a request to raise awareness around the plight of people with physical and visual disabilities (wheelchair and visibility cane emojis).

Within data tagged with demographic markers on Twitter, the majority of people posting and sharing articles about domestic violence in Australia during the 2020 pandemic were female (57.2%), which is not surprising given that women are more likely to be the target of domestic violence [5,18,19,41]. The age of the majority of users tweeting about domestic violence on Twitter specifically were between 25-34 years (43%), followed by those aged 35-44 years (24.7%). Younger women are more likely to be caregivers of children and older relatives, have the responsibility of managing the family budget, and have the added responsibility of homeschooling during the pandemic [11]. They also have greater access to and awareness of social media and technology in general.

Key hashtag drivers of sentiment included words such as “DV,” “MeToo,” “women,” “law” “family law,” “sexism,” “trauma,” “homelessness,” “stress,” “aged care,” and “violence against women.” Interestingly, ageist comments have been prevalent on social media during the COVID-19 pandemic, with posts implying that the lives of older adults are less valuable rising as quickly as 1 day after each news update on increases in COVID-19 infection and death rates or information related to COVID-19 risk factors [71]. The study reported a daily average of ageist tweets of 18% with the highest rate of almost 53% in March 2020. The content of those tweets ranged from suggesting that older people should be isolated to prevent the spread of COVID-19 to death jokes and ridicule targeted at older people.

Social and news media present a unique opportunity to investigate attitudes toward abuse and violence [60,64], and online forums provide an opportunity to offer support, engage in advocacy, and voice concerns or desires for social change [72,73], while also providing a central point for discussion of domestic violence prevention and promotion [60]. This information provides a useful window into the perspectives of persons expressing emotions related to domestic violence. Identifying periods of increased activity on Twitter is a useful way to identify changes in public opinion.

The rise in societal concerns evidenced by negative emotions on social media needs to be monitored regularly by public health professionals, who can release strategic information for consumers, health professionals, and government bodies so that urgent action can be taken during periods of high domestic violence–related activity.

### Limitations

The study was conducted during a pandemic when everyday conditions deviated away from the norm, and use of social and news media was more amplified than in nonpandemic times. It should be noted that while the study was partially based on geotagged posts from across social media posts and articles from other (eg, news) media (in the Australian region), the limitations of geotagged tweets and posts should be kept in mind [74-76]. Drawing on the discussions of the reliability of geotagged research [58], first, while our data sample was quite large, it retained some bias, as users on social media are not representative of the general Australian population. The consequence is that the demographics indicated in this study do not perfectly mirror the larger Australian population. Second, not all users on social and other media platforms are required to allow access to their geotagged location, so, while access was obtained for tens of thousands of geotagged posts, this is still a relatively small proportion of the total of nongeotagged posts across social and other media. However, despite these limitations, our results within this limited data sample have produced insights and metrics that align with existing qualitative trends and research with regards to domestic violence rates and policies within the COVID-19 pandemic [77,78]. Future research will need to add to unstructured social media and news data with analysis from interview or focus group data via traditional qualitative or digital ethnographic methods [52]. Further, domestic violence is a sensitive topic, and the way it is talked about on social media or online news posts may not fully represent all narratives. Hence, domestic violence posts on social media during COVID-19 can only represent the opinions and reactions of current geotagged social media users. In addition, the study focused on posts that included specific search terms and sentiment criteria; future studies would benefit from expanding the search terms used and the social and general media platforms included.

### Conclusion

Social media and news media are important mechanisms for discussing and sharing information about domestic violence and available services. They represent an important avenue for the dissemination of information; information can be widely dispersed and easily accessed by a range of different communities who are often difficult to reach. The study showed that the use of sentiment and discourse analysis to assess social media and news media data is useful in measuring the general mood toward sensitive issues, the public expression of feelings, and sharing of resources in relation to the otherwise personal experience of domestic violence. Heightened awareness of this could help agencies tailor and target messaging to maximize impact.

## Appendix

Multimedia Appendix 1 (A) Boolean search terms for domestic violence and (B) final Boolean (data sample: English-language posts originating within the Australian geographical region; January 1 to December 12, 2020).

Multimedia Appendix 2 Sentiment analysis of domestic violence posts in Australia: definition and context.
