# Supplementary material for: Public Sentiment and Discourse on Domestic Violence During the COVID-19 Pandemic in Australia: Analysis of Social Media Posts
Source: J Med Internet Res. 2021 Oct 1;23(10):e29025. doi: 10.2196/29025 (PMC8489563; doi:10.2196/29025)
Supplement: Multimedia Appendix 1 [file jmir_v23i10e29025_app1.docx]

**Multimedia Appendix 1.** (A) Boolean search terms for domestic violence and (B) final Boolean (data sample: English-language posts originating within the Australian geographical region; January 1 to December 12, 2020).

## Boolean Search terms for Domestic Violence Exploratory Boolean (Data sample: English language posts originating in Australian geographical region. Date range 01.01.2020 – 31.06.2020): (("domestic violence" OR "family violence" OR "domestic abuse" OR "domesticviolence*" OR "domesticabuse*"))

1. **Final Boolean (Data sample: English language posts originating in Australian geographical region. Date range 01.01.2020 – 31.12.2020):**

   (("coronavirus" OR "#coronavirus" OR “corona” OR "Covid*" OR "coronavi*" OR "COVID-19" OR "COVID 19" OR "COVID19" OR "#COVID19" OR "COVID_19" OR "COVID" OR "severe acute respiratory syndrome coronavirus 2" OR "severe acute respiratory syndrome coronavirus 2" OR "2019-nCoV" OR "SARS-CoV-2" OR "2019nCoV") AND ("Sexism" OR "gender bias" OR "domestic violence" OR "family violence" OR "intimate partner violence" OR "bruises" OR "gender violence" OR "domestic abuse" OR "spous* abuse" OR "spous* violence" OR "domesticviolence*" OR "familyviolence*" OR "domesticabuse*" OR "DFV" OR "DV" OR "VAW" OR "violenceagainstwomen*" OR "femicide" OR "sex-based violence"))
