# Supplementary material for: Public Sentiment and Discourse on Domestic Violence During the COVID-19 Pandemic in Australia: Analysis of Social Media Posts
Source: J Med Internet Res. 2021 Oct 1;23(10):e29025. doi: 10.2196/29025 (PMC8489563; doi:10.2196/29025)
Supplement: Multimedia Appendix 2 [file jmir_v23i10e29025_app2.docx]

# **Appendix 2**

**Sentiment analysis of Domestic Violence posts (Australia)**

**Definition & Context:**

- Analysis of changes to discussion activity related to Domestic Violence in Australia – since the beginning of the COVID-19 pandemic.
- Analysis exploring the impact social restrictions on discussion activity related to domestic violence in Australia due to COVID-19
- Analysis of the people’s ability to contact DA support services during periods of social restriction due to social restrictions

## Sentiment analysis of Domestic Violence posts (Australia)

## Positive (P)

- Post communicating overall trust and satisfaction with Public Health/ Community Outreach guidelines and support for measures of reporting/preventing Domestic Violence.
- Posts encourage discussions about Domestic Violence and seeking/acquiring proactive help.
- Post describes overcoming Domestic Violence problems.

## Negative (N)

- Post contains negative attitude/arguments regarding experience of reporting/preventing Domestic Violence.
- Post describes mental health of victims of Domestic Violence (current issues and potential long-term problems)
- Post highlights lack of Domestic Violence support and provisions and contains questions re. the need for Domestic Violence support.
- Post discourages the following of recommended Domestic Violence prevention measures.

## Neutral (NT)

- Post contains no elements of uncertainty, positive or negative content.
- Post contains general statement(s) or link(s) to item(s) (e.g. news articles/papers) with no expression of sentiment.
- Post includes factual commentary about Domestic Violence, but no other sentiment (e.g. overview of Public Health or Community Outreach policies).
